# Clinical outcome of instrumented fusion for the treatment of failed back surgery syndrome: a case series of 100 patients

**DOI:** 10.1007/s00701-012-1380-7

**Published:** 2012-05-16

**Authors:** Mark P. Arts, Nicola I. Kols, Suzanne M. Onderwater, Wilco C. Peul

**Affiliations:** Department of Neurosurgery, Medical Centre Haaglanden, PO Box 432, 2501 CK The Hague, The Netherlands

**Keywords:** Failed back surgery syndrome, Instrumented fusion, Pain, Postlaminectomy syndrome, Surgery

## Abstract

**Object:**

Failed back surgery syndrome is defined as persistent chronic low-back pain and/or leg pain lasting more than 1 year, despite of one or more surgical procedures. Instrumented spinal fusion has been offered by surgeons as a potential treatment to recover from pain and functional disability. Factors contributing to good outcome of instrumented spinal fusion have not been investigated extensively. This study evaluated the global perceived recovery and functional status of patients after instrumented fusion for the treatment of failed back surgery syndrome.

**Methods:**

Between January 2004 and September 2007, 100 patients underwent instrumented spinal fusion because of persistent back and/or leg pain lasting more than 1 year despite of one or more previous spine surgeries. The global perceived recovery of the patients was documented on a seven-point Likert scale, in which good outcome was defined as “complete recovery” and “almost complete recovery”. Pain was evaluated by the 100-mm visual analogue scale (VAS) of back pain and leg pain, and functional disability measured by the Roland Disability Questionnaire for Sciatica (RDQ) and Oswestry Disability Index (ODI). The Hospital Anxiety and Depression Scale (HADS) evaluated psychological co-morbidity. All patients were sent questionnaires by mail. Pearson’s correlation coefficient was calculated between outcome measures and preoperative patient characteristics.

**Results:**

Eighty-two patients (82% response rate) returned questionnaires that were useful for analysis. After a mean follow-up period of 15 months, 35% of the patients reported good outcome, whereas 65% had unsatisfactory outcome. The mean (± SD) score of VAS low-back pain and leg pain was 45.7 ± 29 and 37.9 ± 31.9, respectively. The mean (± SD) RDQ and ODI score was 11.8 ± 5.4 and 30.6 ± 20.3, respectively. HADS score indicated a possible anxiety disorder in 28% of the patients and in 30% a possible underlying depression. Of the patients’ baseline characteristics, there was only a significantly negative correlation between level of education and outcome.

**Conclusions:**

The present study showed disappointing outcome of instrumented fusion for the treatment of failed back surgery syndrome in terms of perceived recovery, functional disability and pain. Conservative management is probably more beneficial and, therefore, more selective and careful assessment should be done in order to prevent unnecessary surgery.

## Introduction

Failed back surgery syndrome (FBSS), also known as post-laminectomy syndrome, is defined as persistent low-back pain and/or leg pain lasting more than 1 year despite one or more surgical procedures. Nearly 20% of the patients undergoing spine surgery will require secondary surgery for persistent pain or surgery related complications during the subsequent years [9]. Success rates have been reported to drop to 30% after a second spine surgery, 15% after the third surgery, and approximately 5% after the fourth surgical intervention [7]. Multiple factors can contribute to the development of FBSS. Surgery-related factors may be wrong surgical technique, surgical complications, instability, recurrent disc herniation, and fibrosis-associated neuropathic pain. Age, lifestyle (i.e. smoking, obesity, lack of physical exercise) and psychosocial factors, such as depression, anxiety and insomnia, are possible patient-related characteristics. All of these factors may negatively influence the results of instrumented spinal fusion [4,6,12,14–16].

The “gold standard” in the treatment of FBSS is conservative management by pain control and cognitive behavioural therapy, although some FBSS patients may benefit from instrumented spinal fusion [4,5,7,15]. Careful assessment by anamnesis, medical history, physical examination, and additional screening tools is crucial to identify possible physical causes of FBSS. Apt patient selection may therefore lead to improved surgical outcome [1–5].

Previous studies regarding clinical outcome of instrumented spinal fusion in patients with FBSS, have not focused on possible associations between outcome and predefined influencing factors [5]. In the present case series of 100 patients with FBSS who underwent instrumented spinal fusion, associations between patient characteristics and outcome were evaluated.

## Methods

### Study design and patient population

The study was performed as a retrospective case series. All patients who had undergone instrumented spinal fusion between January 2004 and September 2007 were selected from the hospital electronic patient system (487 patients). Inclusion criteria were instrumented lumbosacral spinal fusion, one or more previous lumbosacral spine surgeries, and chronic low-back pain with or without leg pain, lasting more than 1 year after the index surgery (Table 1). Pain was refractory to conventional pain medication, although adhesiolysis, spinal cord stimulation (SCS) or epiduroscopy was not performed. From the 487 patients, 100 patients fulfilled the criteria (49 men and 51 women, mean age 54.2 years). Patients’ baseline characteristics are listed in Table 2. All patients underwent pedicle screw fixation with interbody fusion at one level (58 patients), two levels (28 patients), or three or more levels (14 patients). All procedures were performed in Medical Centre Haaglanden by the first and last author (MA and WP).

**Table 1:** Inclusion and exclusion criteria

Inclusion criteria	Exclusion criteria
Instrumented lumbar or lumbosacral fusion	Instrumented cervical or thoracic fusion
History of ≥ 1 lumbosacral spine surgery	Surgery for spinal tumours
Chronic low-back pain, with or without leg pain, lasting ≥1 year after index surgery	Surgery for spinal trauma

**Table 2:** Demographic data of 100 patients with FBSS

Baseline characteristics	No.
Sex	
Male	49
Female	51
Level of education	
Basic vocational education	29
Secondary vocational education	28
Higher vocational education	21
Other	3
Missing	20
Work capability	
Normal	6
Moderate	13
Nearly incapable	2
Completely incapable	21
NA	40
Missing	18
Smoking	35
Mean Body Mass Index (SD)	26.8 (4.2)
Mean age at surgery in years (SD)	54.2 (12.0)
Etiology of FBSS	
Previous discectomy	41
Previous laminectomy	18
Adjacent level disease after instrumented fusion	21
Instability	15
Other	5
No. of previous surgeries	
1	59
2	20
3	12
4	7
5	2

*NA* not applicable, *SD* standard deviation

### Outcome measures

The primary outcome measure was the dichotomised Likert score in good recovery (“complete recovery” and “almost complete recovery”) and bad recovery (“little recovery” to “worse than ever”). Secondary outcome measures were the postoperative scores of the visual analogue scale (VAS) of low-back pain (0-100 mm) and VAS leg pain (0-100 mm). Functional assessment was documented by the illness-specific Roland Disability Questionnaire for Sciatica (RDQ) (0–23 with higher scores indicating worse functional status) and Oswestry Disability Index (ODI) (0–100 with higher index indicating worse disability). Assessment of anxiety or depression was documented by the Hospital Anxiety and Depression Scale (HADS). The HADS consists of a seven-item depression scale and a seven-item anxiety scale. The score ranges from 0–21 with a high score being indicative for depression/anxiety. The outcomes were assessed by a written questionnaire sent by mail.

### Data analysis

All statistical analyses were performed by SPSS version 17.0 for Windows. Per included patient, the RDQ, ODI, HADS-anxiety and HADS-depression scores were calculated. Pearson’s correlation coefficients were calculated to analyse the association between the different outcome measures and preoperative patient characteristics (gender, level of education, age at surgery, body mass index, smoking status, number of previous surgeries and number of spinal levels fused). All statistical analyses had a statistical threshold of *p* = 0.05.

## Results

Of the 100 questionnaires sent by mail, two respondents had only filled in remarks on the questionnaire and 16 patients were lost to follow-up despite several attempts of telephone and mail contact. These 18 patients were excluded from analysis. The mean follow-up was 14.7 months (min. 2 months, max. 45 months). Of the responding 82 patients, 35% documented good recovery and 65% perceived no difference or worsening. The mean (± SD) score of VAS back pain and leg pain was 45.7 ± 29.6 and 37.9 ± 31.9, respectively. Functional disability measured by RDQ and ODI was 11.8 ± 5.4 and 30.6 ± 20.3, respectively. Assessment of the HADS documented that 28% had symptoms befitting an underlying anxiety disorder, and 30% showed symptoms related to an underlying depression (HADS scores ≥ 8) (Table 3). A significantly negative correlation was only found between patients’ perceived recovery and level of education (*R* = −0.228; *p* < 0.05). Other correlations between perceived recovery and preoperative characteristics could not be found

**Table 3:** Postoperative assessment of outcome measures of 82 respondents with FBBS

Outcome measure	No. (%)
Likert	
Good recovery	29 (35)
Bad recovery	43 (65)
Mean VAS low-back pain (mm)	45.7 ± 29.6
VAS low-back pain	
< 40 mm	33 (40)
≥ 40 mm	49 (60)
Mean VAS leg pain (mm)	37.9 ± 31.9
VAS leg pain	
< 40	46 (56)
≥ 40	36 (44)
Mean RDQ	11.8 ± 5.4
RDQ < 8	17 (21)
RDQ ≥ 8	65 (79)
Mean ODI (%)	30.6 ± 20.3
ODI < 25% disability	29 (35)
ODI ≥ 25% disability	53 (65)
HADS anxiety	
< 8	59 (72)
≥ 8	23 (28)
HADS depression	
< 8	57 (70)
≥ 8	25 (30)

## Discussion

The present study shows that the outcome of instrumented spinal fusion in patients with FBSS is disappointing; only 35% of the patients reported good outcome, whereas in 65% the symptoms were unchanged or worse. Assessment of postoperative pain and functional disability showed that the majority of the respondents experienced moderate to severe pain and functional disability following spinal fusion. In addition, nearly one-third of the respondents had an indication of an anxiety or depression disorder, which correlated with predominantly back pain and functional disability.

The number of patients with FBSS is increasing as more spine surgeries are being performed and many are not successful. Preoperative counselling on patient expectations and surgical success are vital to achieve a realistic goal for both surgeon and patient. Since multiple factors may contribute to the development of FBSS, it is of utmost importance to carefully assess the patient’s history and medical records of the previous surgeries on psychological risk factors, previous treatment regimes for chronic pain, significant pathology not addressed during earlier consultations, and incorrect diagnosis. Psychological risk factors need attention prior to and directly after surgery but should not exclude a patient with a clear indication for surgery. Possible biomechanical causes of persistent pain, such as poor posture, pes planus, and leg-length difference may also contribute to chronic low-back pain [13].

Inappropriate surgical technique of the index operation may result in spinal instability or adjacent-level disease several years later. According to a retrospective study, the incidence of spinal instability will increase from 12% after one surgery, to 50% after four or more revision surgeries [7]. Exploration at the wrong level, as well as surgeon’s inability to achieve the surgical goal, may result in persisting pain. Progression of degenerative disease at the index level or adjacent level, such as imbalanced weight distribution on the facet joints, may cause further stenosis and pain. In addition, surgical complications may arise and can involve cerebrospinal fluid leakage, wound infection, nerve root injury, epidural hematomas, arachnoiditis, pseudomeningocele and battered root syndrome [3]. Evaluating these features should improve patient selection for spinal fusion or alternative treatment options.

Currently, the gold standard in the treatment of FBSS involves conservative management. Pharmacological treatment, such as antidepressants, NSAIDs, COX-2 inhibitors, tramadol, opioids, muscle relaxants and gabapentinoids, is prescribed to reduce pain and facilitate physical activity. When conservative management fails, pain intervention such as medial branch block, epidural steroids, percutaneous epidural adhesiolysis, intrathecal drug administration, and spinal cord stimulation (SCS) need to be evaluated. Preferably, these approaches should be discussed by an interdisciplinary management team [3].

Evidence-based guidelines on various treatment regimes of patients with FBSS are lacking since high-quality randomised controlled trials on this topic are scarce. Brox et al. [2] compared the effectiveness of lumbar fusion with cognitive intervention and exercise in 60 patients. Both groups reported 50% success rates and lumbar fusion did not show any superiority. Kumar et al. [8] randomised 100 patients with FBSS in conventional pain medication management with or without spinal cord stimulation. Patients treated with additional SCS reported better pain relief, functional ability and patient satisfaction. However, due to the large number of crossovers, the results should be interpreted carefully. North et al. [11] randomised 50 patients with FBSS in reoperation and SCS. Similarly, it was found that significantly more patients from the reoperation group crossed over to the SCS group. Patient satisfaction and pain control were significantly higher in the SCS group, both randomised and crossover, making SCS more successful than reoperation. In general, revision surgery should be evaluated carefully, as the overall success rate in patients with FBSS following previous spine surgery is low and declining with each subsequent procedure. Follow-up rates up to 5 years documented a good outcome between 19% and 34%, where success was defined by the criterion of at least 50% pain relief [10,11]. The decision to proceed with additional revision surgery, however, was based on the surgeon’s assessment of the patient.

Regarding demographic data and outcome in patients with FBSS, it is shown that female gender, higher age, obesity, number of spine surgeries, multiple fused spinal levels, smoking status and high usage of analgesics negatively influence experienced recovery [1,4,6,12,14–16]. Aside from a negative association between perceived recovery and the level of education, no significant relation between perceived recovery and functional disability and baseline characteristics was found in the present study. Our findings are in agreement with the socio-demographical findings reported by the Swedish Lumbar Spine Study, in which influence of personality traits on surgical outcome was investigated. It was shown that patients with neurotic personality, having overlapping characteristics with the dysfunctional personality type, is predictable for an unfavourable outcome and could possibly benefit more from conservative treatment [9]. The relation between outcome and a possible underlying anxiety disorder or depression showed a negative correlation between perceived recovery and functional disability. Notably, a possible underlying depression and the amount of back pain and functional disability assessed by the RDQ showed an association indicating a predictive value for HADS depression, back pain and functional disability. However, based on a single postoperative measurement, no conclusive statements can be made regarding the predictive value of these patient characteristics. Further studies are needed to evaluate the correlation between possible psychiatric co-morbidity and outcome measures.

The present results have several limitations. Firstly, the study is conducted as a single-centre retrospective cohort with limited follow-up moments. Secondly, there is no available data on co-morbidity like osteoporosis, arthritis or polyneuropathy that may have influenced the outcome. Moreover, there is no routine radiographic examination focusing on fusion status, although the relationship between fusion and outcome is debatable [5]. Finally, based on clinical expertise during the course of the cohort, selection bias may have played a role since latter patients may have been evaluated more critically. Future randomised controlled trials on patients with FBSS are needed in order to define the best treatment strategy.

## Conclusions

The present study shows disappointing results in patients with FBSS treated with instrumented spinal fusion. Surgical candidates should be assessed and selected more carefully in order to prevent unnecessary surgery. Therefore, conservative pain management, behavioural therapy, and SCS is probably more beneficial in the majority of patients with FBSS.
